# HPV16-Positive Pelvic Nodal Squamous Cell Carcinoma with No Detectable Cervical Malignancy

**DOI:** 10.3390/diagnostics16050787

**Published:** 2026-03-06

**Authors:** In Sun Hwang, Su Jeong Lee, Chan Joo Kim, Jin-Hwi Kim, Kwangil Yim

**Affiliations:** 1 Department of Obstetrics and Gynecology, Uijeongbu St. Mary’s Hospital, College of Medicine, The Catholic University of Korea, Seoul 11765, Republic of Korea; 2 Department of Hospital Pathology, Uijeongbu St. Mary’s Hospital, College of Medicine, The Catholic University of Korea, Seoul 11765, Republic of Korea

**Keywords:** Uterine Cervical Neoplasms, carcinoma, squamous cell, papillomavirus, Infections, Neoplasms, unknown primary, spontaneous regression

## Abstract

Isolated pelvic nodal metastasis from carcinoma of unknown primary origin (CUP) is rare. Evaluation should prioritize gynecological and anorectal sites based on pelvic lymphatic drainage. Although spontaneous regression of these primary lesions is exceptional, regressed lesions can present as CUP, necessitating diagnostic caution. Here, we report the case of a 40-year-old woman with a solitary, intensely fluorodeoxyglucose F-18 avid left obturator lymph node and a subtle endocervical abnormality on pelvic magnetic resonance imaging. Loop electrosurgical excision revealed a Nabothian cyst only. Excisional nodal biopsy by polymerase chain reaction revealed metastatic squamous cell carcinoma with diffuse block-type p16 and human papillomavirus (HPV) 16. Considering the potential for a primary cervical tumor along the obturator drainage pathway, the patient underwent hysterectomy with pelvic lymph node dissection. No residual invasive carcinoma was found; however, HPV16 was detected in the cervix with a low-grade squamous intraepithelial lesion, supporting a regressed cervical focus. She received adjuvant cisplatin-based chemoradiotherapy and has remained disease-free for 56 months. This case highlights the diagnostic value of integrating lymphatic anatomy with the molecular profile of HPV. Cervical squamous cell carcinoma rarely regresses and presents solely as an isolated nodal disease.

**Figure 1:**
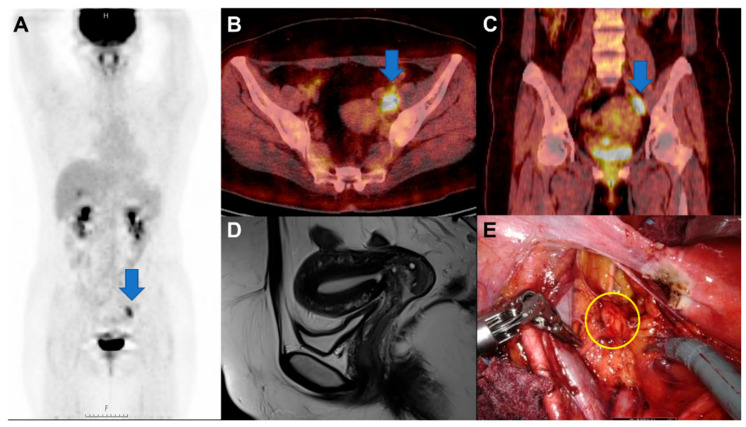
A solitary fluorodeoxyglucose F-18 (^18^F-FDG)-avid obturator node with a subtle cervical lesion. (**A**) This whole-body ^18^F-FDG positron emission tomography/computed tomography (PET/CT) image shows no abnormal hypermetabolic uptake, excluding focal nodular uptake in the left obturator region (arrow). H, head; F, foot. (**B**,**C**) Axial and coronal pelvic PET/CT fusion images demonstrate a hypermetabolic lymph node in the left obturator area (arrows, SUVmax 9.1), raising suspicion for metastatic lymph node involvement from an unknown primary tumor or malignant lymphoma. (**D**) Fat-suppressed T1-weighted magnetic resonance imaging (MRI) in the sagittal plane of the uterus reveals an ill-defined enhancing lesion measuring approximately 1.9 cm in the anterior endocervix, with several cystic components. Although a Nabothian cyst was suspected, gastric-type endocervical adenocarcinoma could not be excluded, considering the hypermetabolic lymph node. A diagnostic loop electrosurgical excision procedure (LEEP) is performed; however, no malignancy was identified in the cervix. (**E**) Laparoscopic exploration demonstrates an enlarged left obturator lymph node (circle). Here, we report the case of a 40-year-old woman (gravida 2, para 2) with two previous cesarean deliveries who presented with upper abdominal pain and was diagnosed with acute calculous cholecystitis. Preoperative abdominal CT incidentally revealed left obturator lymphadenopathy. She underwent laparoscopic cholecystectomy, and pathological examination confirmed acute cholecystitis with cholelithiasis. Considering the unexpected nodal finding suspicious for malignancy, further evaluation was initiated. On day 4, pelvic MRI was obtained, followed by a diagnostic LEEP demonstrating only a Nabothian cyst without malignancy. At three weeks, colonoscopy was performed and no abnormalities were observed in the anus or rectum. At one month, ^18^F-FDG PET/CT confirmed persistent FDG avidity in the left obturator node. Because a focal residual malignancy could not be definitively excluded after the previous excisional procedure, the patient underwent laparoscopic radical hysterectomy with pelvic lymph node dissection for definitive tissue evaluation and gynecologic primary and curative-intent resection. The histopathologic evaluation of the left obturator lymph node revealed squamous cell carcinoma (SCC) with diffuse p16 positivity and human papillomavirus (HPV) 16 positivity. Based on comprehensive clinical, radiologic, and surgical findings, the tumor origin was determined to originate from the uterine cervix. Pathologic involvement of the obturator lymph node corresponded to stage IIIC1p cervical cancer, for which guideline-recommended concurrent chemoradiation therapy (CCRT) was subsequently administered as standard-of-care treatment [1].

**Figure 2:**
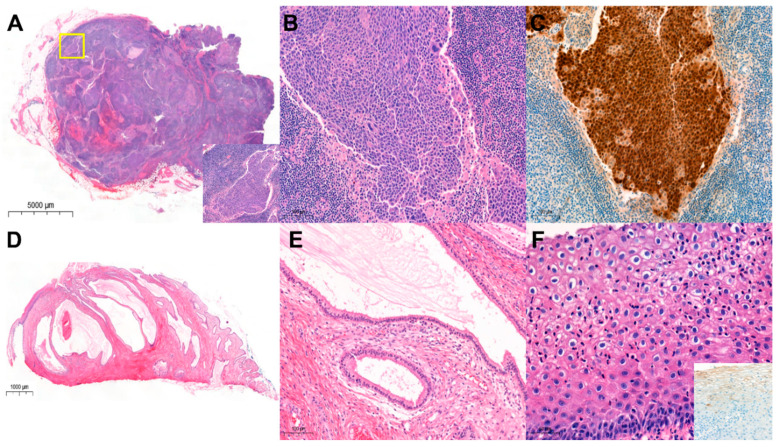
Histological (**A**,**B**,**D**–**F**) and p16 immunohistochemical (**C**) findings of obturator lymph nodes (**A**–**C**) and uterine cervix (**D**–**F**). An enlarged obturator lymph node shows solid sheets of carcinoma cells within the lymph node sinus (**A**, scanning view). The boxed area in A is shown at higher magnification in the inset (×100). In high-power view, the tumor shows basaloid features with squamous differentiation, consistent with metastatic SCC (**B**, ×200). Scale bar = 100 µm. Immunohistochemistry shows diffuse block-positive p16 (**C**, ×200) (Scale bar = 100 µm) and focal cytokeratin positivity, whereas cytokeratin 20, CDX2, PAX2, PAX8, and GATA3 are negative. Real-time polymerase chain reaction (PCR) for HPV performed on the nodal tissue detects HPV16. In the uterine cervix, dilated endocervical glands are noted (**D**, scanning view). On high-power magnification, the cyst is lined by cytological bland cells (**E**, ×200) Scale bar = 100 µm. The Ki-67 index is <1%, p53 shows a wild-type pattern, PAX2 expression is preserved, and MUC6 is negative. These findings are consistent with a diagnosis of a Nabothian cyst. In the exocervix, neither SCC nor a high-grade squamous intraepithelial lesion is seen; however, koilocytosis consistent with a low-grade squamous intraepithelial lesion (LSIL) is present (**F**, ×400). Scale bar = 50 µm. The real-time PCR detects HPV16, but p16 immunostaining shows only patchy staining (inset of **F**, ×200).

**Figure 3:**
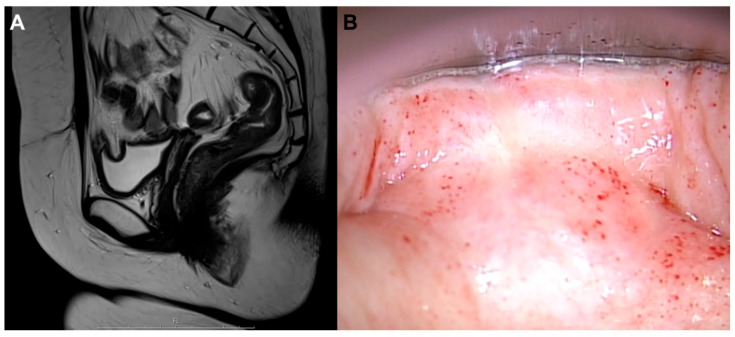
Follow-up sagittal pelvic MRI after radical hysterectomy followed by six cycles of concurrent chemoradiotherapy shows no evidence of residual or recurrent mass in the pelvic cavity. F; foot. (**A**). Follow-up colposcopy demonstrates a normal-appearing vaginal vault without any suspicious lesion, consistent with no evidence of disease (**B**). The patient underwent postoperative concurrent cisplatin-based chemoradiotherapy with pelvic radiotherapy at 45 Gy in 25 fractions and a boost to the involved nodal bed for 60–62.5 Gy. Posttreatment surveillance, including cervical cytology and serial imaging, demonstrated a complete clinical and radiologic response, and the patient has remained alive and disease-free for 56 months. Carcinoma of unknown primary origin (CUP) is a metastatic malignancy in which the primary site remains unidentified after a standardized diagnostic evaluation. Since the management of CUP is largely driven by histology and immunophenotypes, obtaining adequate tissue for immunohistochemical evaluation is pivotal [1,2,3,4]. In patients with isolated pelvic nodal disease, site-directed evaluation should be guided by typical lymphatic drainage, focusing on the gynecological and anorectal primaries that commonly metastasize to the internal iliac and obturator nodes [5,6]. Histologically, CUP can be classified into four major subtypes: SCC, adenocarcinoma, poorly differentiated carcinoma, and neuroendocrine carcinoma. Among these, SCC-type CUP accounts for approximately 5–15% of cases and presents as lymph node metastases with characteristic anatomical distributions, including the mediastinal–retroperitoneal, axillary, cervical, and inguinal regions [1,2,3,4]. Cervical lymphadenopathy frequently suggests an occult head and neck primary tumor and may benefit from PET/CT imaging, whereas inguinal involvement raises the possibility of an anorectal, urogenital, or cutaneous origin, requiring the exclusion of melanoma and lymphoma [5,6]. Presently, HPV was detected in the metastatic SCC, supporting an HPV-associated tumor. High-risk HPV-associated SCC most commonly arises in the uterine cervix but may originate from other anogenital sites or the head and neck region through E6/E7-mediated disruption of the p53 and Rb pathways [7,8,9]. In this case, the disease was confined to a solitary obturator lymph node without cervical chain involvement. This distribution is more consistent with anogenital lymphatic drainage than the typical nodal spread of head and neck SCC (HNSCC), predominantly involving the upper and midcervical levels [4,10]. Although distant intra-abdominal or pelvic nodal dissemination from HNSCC has been reported, it is rare and usually occurs in patients with advanced primary or recurrent disease [11,12,13,14]. Therefore, in the absence of a detectable primary head and neck tumor, an occult gynecological or anorectal origin was prioritized in the subsequent workup. However, in similar clinicopathologic settings, HPV-associated pelvic or retroperitoneal SCC is often considered primary retroperitoneal disease or classified as CUP when no mucosal origin is identified (Table A1) [15,16,17,18,19,20,21,22,23,24]. Only three cases (including ours) have reported identification of the primary site [17,18]. In all three, the tumor was managed as originating from the anogenital area, and treatment followed the corresponding guideline-based approach [17,18]. The present case was presumed to be of cervical origin with obturator lymph node metastasis (regional nodal disease) and was therefore staged as FIGO 2018 IIIC and treated with definitive CCRT. Pai et al. similarly presumed a cervical primary, but superficial inguinal nodal involvement represents non-regional disease in cervical cancer and is considered distant metastasis; accordingly, their case was managed as stage IV with systemic chemotherapy [17]. By contrast, Gulvin et al. suspected an anal canal primary, in which groin nodes are regional; their case was thus treated as stage III with definitive CCRT [18]. These cases illustrate that the same lymph node involvement can be staged differently by tumor type: inguinal lymph nodes are considered distant metastasis in cervical cancer but regional nodal disease in anal cancer. As a result, when the primary site can be estimated, similar nodal findings may lead to different stage assignments and different standard-of-care treatments. In contrast, when the primary site could not be identified, no standardized treatment strategy was available, and management was individualized according to each clinical context (Table A1) [15,16,19,20,21,22,23,24]. Across the reported cases, some appeared to receive more aggressive treatment [15,16,19,20,21,22], whereas others underwent less intensive or palliative treatment [16,19,23,24]. Although follow-up was generally short and varied widely, a rough comparison suggests that 8 of 9 patients treated aggressively achieved no evidence of disease (NED), while only 1 of 6 patients treated with less intensive or palliative approaches achieved NED. This pattern suggests that even when the primary site is not found or is suspected to have regressed; it may be beneficial to consider guideline-based treatment targeting the most likely primary site, when feasible. Nevertheless, if the primary site cannot be determined, it is inherently difficult to design the most appropriate treatment. Therefore, identifying the primary site should remain a priority whenever possible. HPV- or p16-positive isolated pelvic nodal SCC is rare, and standardized diagnostic and therapeutic strategies remain undefined when the primary site cannot be identified. Consistent with NCCN guideline recommendations for CUP, we performed a comprehensive diagnostic evaluation, including detailed clinical examination, imaging studies, and pathologic assessment to identify a potential mucosal primary site. Rather than relying solely on imaging-based evaluation or histopathologic confirmation, a multidisciplinary approach integrating clinical, radiologic, pathologic, and surgical findings was adopted. Although no standardized algorithm defines the priority or sequence of diagnostic evaluation in HPV-associated pelvic nodal SCC, assessment should be individualized according to the clinical context. Nevertheless, an extensive guideline-based workup remains essential, incorporating repeated imaging assessment, careful evaluation of pelvic mucosal sites, and multidisciplinary decision-making [15,16,20]. However, when a Papanicolaou smear or biopsy of the cervix or anus does not reveal a lesion, prophylactic total resection is not recommended, because it is overly invasive. In the present case, we took a multidisciplinary approach. A cervical lesion was suspected on MRI, but conization was interpreted as a Nabothian cyst. Because a deeply located occult malignancy could not be fully excluded, total hysterectomy was performed, and focal residual LSIL was identified in the cervix. This case suggests that routine screening may fail to detect the primary lesion, and that a primary in the cervix may be inferred only incidentally during evaluation of a coexisting cystic lesion. This also helps explain why cases with regressed primary lesions are often labeled clinically as carcinoma of unknown primary or as a “primary” retroperitoneal malignancy. Cases of presumed primary tumor regression with subsequent retroperitoneal metastasis have been reported, in which the primary was presumed to originate from the uterine cervix [17] or anogenital area [18]. However, in those reports, the primary lesion was either not evaluated [17] or only a limited biopsy was performed [18]; therefore, a comprehensive assessment of the primary site was not achieved. Furthermore, HPV infection has not been documented in tumors in previously reported cases [17,18]. Presently, LSIL was identified in a hysterectomy specimen, and HPV16 was detected in the metastatic tumor and uterine cervix, suggesting regression of uterine cervical SCC. Contrasting with disseminated CUP, which typically has a poor prognosis [2,25], this case followed a localized course with a favorable outcome, supporting the possibility of spontaneously regressed uterine cervical carcinoma rather than conventional CUP [26]. Spontaneous regression is well documented in cervical intraepithelial neoplasia (CIN); for example, approximately 15–20% of CIN3 lesions may regress if the interval between biopsy and conization is prolonged [27,28]. Although spontaneous regression of invasive uterine cervical carcinoma has been reported only rarely [29], regression of malignant tumors in general is a recognized phenomenon and has been attributed to immune-mediated mechanisms and other host-related factors [30]. In HPV-associated squamous malignancies, spontaneous regression has been analyzed in HNSCC, where host immune activation appears to contribute to tumor control. In that analysis, immune-mediated regression was categorized into three principal mechanisms: biopsy-induced micro-trauma leading to enhanced antigen exposure and cytotoxic T-cell activation; febrile illness and hyperthermia-associated immune stimulation; and microbial or oral microbiome-driven cytokine activation [31]. In the present case, the patient presented with acute cholecystitis accompanied by high fever exceeding 38 °C prior to the diagnosis of malignancy. Acute inflammatory states with fever are known to enhance antigen presentation and cytotoxic immune responses, including activation of CD8+ T cells and natural killer cells, as well as upregulation of heat-shock proteins and MHC class I expression. Although causality cannot be established, the temporal proximity between the febrile inflammatory episode and subsequent regression of the cervical lesion raises the possibility that systemic immune activation may have contributed to the observed spontaneous regression. A previously reported case of spontaneous regression in cervical adenocarcinoma was interpreted as a radiation-induced abscopal effect following prior radiotherapy [32]. In contrast, our patient had not received any oncologic treatment before tumor regression, suggesting a distinct immune-triggering event. This distinction may indicate that spontaneous regression in cervical carcinoma cancer occurs through heterogeneous immunologic pathways, including treatment-induced and non-treatment-related systemic immune activation. This case underscores the rarity and biological interest in HPV-associated malignancies presenting with isolated nodal metastasis despite the absence of a detectable primary lesion, raising the possibility that uterine cervical SCC may undergo spontaneous regression. Integration of PET/CT findings with detailed histopathologic and virologic evaluation was pivotal in narrowing the differential diagnosis and is consistent with the concept of a vanishing cervical primary. Importantly, this case highlights a key clinical lesson: when isolated pelvic lymph node metastasis is encountered, a regressed uterine cervical carcinoma should be actively considered, and it offers a practical lesson for treatment decisions, particularly regarding whether to apply guideline-based therapy directed at the most likely primary site. While rare reports have described pelvic nodal metastases arising from regressed anorectal [18] or uterine cervix primaries [17], those cases lacked definitive corroborating evidence. In contrast, our case demonstrated convergent findings; absence of another primary on PET/CT, detection of HPV16 within the metastatic tumor, and LSIL identified on a hysterectomy specimen. Taken together, these findings were most compatible with metastatic disease arising from a regressed cervical carcinoma.

## Data Availability

No datasets were generated or analyzed during this study.

## Abbreviations

The following abbreviations are used in this manuscript:

CIN	Cervical Intraepithelial Neoplasia
CT	Computed Tomography
CUP	Carcinoma of Unknown Primary
FDG	Fluorodeoxyglucose
HNSCC	Head and Neck Squamous Cell Carcinoma
HPV	Human Papilloma Virus
LEEP	Loop Electrosurgical Excision Procedure
LSIL	Low-grade Squamous Intraepithelial Lesion
MRI	Magnetic resonance imaging
NED	No Evidence of Disease
PET	Positron Emission Tomography
PCR	Polymerase Chain Reaction
SCC	Squamous Cell Carcinoma

## Appendix A

**Table A1:** Previously reported cases of pelvic or retroperitoneal squamous cell carcinoma and comparison with the present case.

Primary Site Determination	Case	Authors	Patient Age	Location of Mass	HPV/p16 IHC	Primary Site	Metastatic Disease	Initial Treatment	Clinical Outcome
Identified	1	Current case	40/F	Lt. obturator lymph node	HPV+/p16+	Cervix	Solitary disease	Surgical resection followed CCRT (cisplatin)	NED at 56 months
	2	Pai et al. [17] 2016	35/F	Rt. superficial inguinal lymph node	HPV unknown/p16 unknown	Cervix	Advanced disease	Systemic therapy (6 cycles of carboplatin plus paclitaxel)	NED at 3 months
	3	Gulvin et al. [18] 2016	53/F	Lt. groin node	HPV+/p16+	Anus	Solitary metastasis	Radiation therapy followed by systemic therapy (5-fluorauracil plus mitomycin C)	NED at 18 months
Not identified	4	Clements et al. [19], 2010	34/F	Rt. psoas	HPV−/p16+	Retroperitoneum	Solitary disease	CCRT (cisplatin)	Stable disease for 1 followed by progression. Alive with disease at 23 months.
	5	Clements et al. [19], 2010	54/F	Rt. pelvic lymph node	HPV+/p16+	Retroperitoneum	Solitary disease	Surgical resection followed by CCRT (carboplatin)	NED at 48 months
	6	Clements et al. [19], 2010	44/F	Lt. psoas	HPV+/p16+	Retroperitoneum	Solitary disease	Systemic therapy followed by CCRT	NED at 6 months
	7	Clements et al. [19], 2010	27/F	Lt. psoas	HPV unknown/p16+	Retroperitoneum	Solitary disease	Neoadjuvant chemotherapy with local progression, followed by CCRT	Died 12 months after diagnosis
	8	Clements et al. [19], 2010	43/F	Lt. psoas	HPV+/p16+	Retroperitoneum	Solitary disease	Palliative radiation	Progression at 12 months in primary tumor and regional lymphatics
	9	Clements et al. [19], 2010	52/F	Rt. psoas	HPV+/p16+	Retroperitoneum	Distant metastasis	Palliative radiation	Died 8 months after diagnosis
	10	Isbell et al. [16], 2016	69/F	Lt. retroperitoneum	HPV+/p16+	Retroperitoneum	Solitary disease	CCRT (cisplatin)	NED at 7 months
	11	Isbell et al. [16], 2016	58/F	Lt. pelvic wall	HPV unknown/p16+	Retroperitoneum	Solitary disease	CCRT (cisplatin)	NED at 48months
	12	Isbell et al. [16], 2016	47/F	Lt. retroperitoneum	HPV unknown/p16+	Retroperitoneum	Solitary disease	Patient declined treatment	Died 12 months after diagnosis
	13	Matsuzaka et al. [20], 2019	76/F	Lt. adenxa	HPV+/p16+	Retroperitoneum	Advanced disease	Surgical resection followed by systemic therapy (carboplatin plus paclitaxel)	NED at 6 months
	14	Cucinella et al. [21], 2022	52/F	Retroperitoneal para-rectal mass	HPV−/p16+	Retroperitoneum	Solitary disease	Surgical resection followed by systemic therapy (6 cycles of carboplatin plus paclitaxel)	NED at 6 months
	15	Zhu et al. [22], 2023	43/F	Rt. adenxa	HPV unknown/p16+	Retroperitoneum	Advanced metastasis	Surgical resection followed by systemic therapy (carboplatin plus paclitaxel)	NED at 72 months
	16	Matylevich et al. [23], 2024	62/F	Pelvic floor muscle	HPV+/p16+	Retroperitoneum	Solitary disease	Surgical resection of pelvic floor mass, no adjuvant therapy	NED at 16 months
	17	Koge et al. [24], 2024	64/F	Pelvis	HPV unknown/p16+	Retroperitoneum	Solitary disease	Palliative radiation	Progression-free survival for 7 months
	18	Dubey et al. [15], 2025	69/F	Pre-sacral mass	HPV+/p16+	Retroperitoneum	Distant metastasis	Systemic therapy (Carboplatin, paclitaxel, pembrolizumab, bevacizumab)	NED at 23 months of follow-up
